# From 2022 to 2025: Preventing the Escalating Health Crisis of Pakistan’s Floods

**DOI:** 10.5334/aogh.4955

**Published:** 2025-12-10

**Authors:** Rehan Jan, Maryam Jan, Noman Jan

**Affiliations:** 1 Federal Medical College, Shaheed Zulfiqar Ali Bhutto Medical University (SZABMU), Islamabad, Pakistan; 2 Department of Human Nutrition and Dietetics, Ziauddin University, Karachi, Pakistan; 3 Bolan Medical College, Quetta, Pakistan

In 2022, unprecedented monsoon rains submerged nearly one‑third of Pakistan, displacing more than 33 million people and destroying more than 2,000 health facilities [1]. The floods represented one of the country’s worst disasters in recent history. Food insecurity surged as crops and livestock were washed away, leaving an estimated 8.6 million people in need of urgent nutrition assistance [1]. With child malnutrition already widespread, the destruction of food supplies placed millions of children at increased risk of stunting and wasting. Scientific analysis by the World Weather Attribution initiative confirms that climate change likely intensified these rains, exacerbating the severity of this humanitarian crisis [2].

The collapse of water, sanitation, and hygiene (WASH) infrastructure compounded the crisis, triggering predictable outbreaks of diarrheal diseases, malaria, and dengue that overwhelmed limited health services [3]. Respiratory infections, skin diseases, and snakebites were also widely reported in overcrowded shelters, reflecting how displacement magnifies health risks when basic services are absent [3]. However, accurately quantifying this health burden remains challenging. The destruction of reporting infrastructure in submerged districts resulted in fragmented surveillance data, particularly regarding noncommunicable diseases and mental health. This data gap obscures the full extent of the crisis and hinders precise resource allocation.

Women and girls bore a disproportionate burden. Disrupted access to antenatal care, contraception, and safe delivery services heightened the risks of maternal morbidity and mortality [4]. In many affected areas, reproductive health services were absent or deprioritized, forcing women to give birth in unsafe conditions [4, 5]. The absence of privacy, protection, and continuity of care in emergency shelters further increased the risks of gender‑based violence [5]. These inequities demonstrate that sexual and reproductive health must be treated as an essential, non‑negotiable part of disaster response. However, vulnerability extended beyond gender. The disaster deepened the rural–urban health divide, with remote agrarian communities facing prolonged isolation from relief efforts compared to accessible urban centers. Additionally, the elderly and persons with disabilities encountered severe barriers in accessing evacuation routes and mobile health units, highlighting a critical gap in inclusive disaster planning.

The institutional response to the 2022 floods was marked by delayed mobilization, fragmented governance, and weak coordination across federal, provincial, and humanitarian actors [6]. Relief efforts were hampered by inequitable allocation of resources and poor integration of community voices, leaving the most vulnerable—children, women, and the elderly—underserved [6]. Unless these governance gaps are addressed, Pakistan will remain ill‑prepared for future disasters of similar or greater scale.

That future has already arrived. In August 2025, Pakistan is once again experiencing devastating floods, a stark reminder of how recurrent and predictable such events have become. Without urgent enforcement of disaster policies and investment in resilience, outcomes could be even more catastrophic than in 2022. This is not simply a humanitarian challenge but a test of political will and governance.

Building climate‑resilient health systems is now an urgent imperative [7]. The Ministry of National Health Services, Regulations and Coordination must prioritize strengthening primary health care, with investment in community‑based surveillance, secure supply chains for essential medicines, and enhanced capacity for frontline workers [7]. At the same time, provincial health departments must lead nutrition programs targeting pregnant women and young children to mitigate long‑term intergenerational health impacts. The National Disaster Management Authority must ensure that robust WASH infrastructure and climate‑resilient agriculture are integrated into preparedness plans [6]. Civil society and local communities should be empowered as partners in planning and recovery to ensure accountability and inclusivity.

The 2022 floods were a turning point. The renewed devastation in August 2025 underscores that health is not a downstream casualty of climate disasters but a domain where resilience can—and must—be built. Unless Pakistan decisively invests in climate‑adaptive health systems and enforces evidence‑based disaster policies, the next flood will not only replicate but surpass past tragedies. The window for action is rapidly closing.

## References

[r1] Devi S. Pakistan floods: Impact on food security and health systems. Lancet. September 10, 2022;400(10353):799–800. PMID: . 36088939 10.1016/S0140-6736(22)01732-9

[r2] World Weather Attribution. Climate change likely intensified heavy monsoon rain in Pakistan, exacerbating urban floods that impacted highly exposed communities [Internet]. 2025. Accessed November 22, 2025. https://www.worldweatherattribution.org/climate-change-likely-intensified-heavy-monsoon-rain-in-pakistan-exacerbating-urban-floods-that-impacted-highly-exposed-communities/.

[r3] Alied M, Salam A, Sediqi SM, Kwaah PA, Tran L, Huy NT. Disaster after disaster: The outbreak of infectious diseases in Pakistan in the wake of 2022 floods. Ann Med Surg (Lond). December 8, 2023;86(2):891–898. PMID: . 38333326 10.1097/MS9.0000000000001597 PMC10849431

[r4] Waseem S, Ahmed SH, Ahmed KAHM, Shaikh TG, Ullah I. Reproductive health crisis amidst a natural disaster in Pakistan: A call to action. Womens Health (Lond). 2025;21:17455057251344725. PMID: . 40478606 10.1177/17455057251344725 PMC12144374

[r5] Ashraf M, Shahzad S, Sequeria P, Bashir A, Azmat SK. Understanding challenges women face in flood‑affected areas to access sexual and reproductive health services: A rapid assessment from a disaster‑torn Pakistan. Biomed Res Int. April 1, 2024; 2024:1113634. PMID: . 38590384 10.1155/2024/1113634 PMC11001467

[r6] Abdullah MA, Shaikh BT, Sikander A, Sarwar B. Public health and health system’s responsiveness during the 2022 floods in Pakistan: What needs to be done? Disaster Med Public Health Prep. January 2, 2024;17:e567. PMID: . 38163991 10.1017/dmp.2023.224

[r7] Akthar TM, Reid MJA. The urgency of climate‑resilient health systems in Pakistan: Lessons from the 2022 floods. Int J Public Health. November 1, 2024;69:1607981. doi:10.3389/ijph.2024.1607981. 39545056 PMC11563755

